# Surfers' knots in the anterior chest

**DOI:** 10.1002/ccr3.2048

**Published:** 2019-02-12

**Authors:** Akira Baba, Yumi Okuyama, Hiroyuki Yakabe, Shinji Yamazoe, Yuko Kobashi, Takuji Mogami

**Affiliations:** ^1^ Department of Radiology The Jikei University School of Medicine Tokyo Japan; ^2^ Department of Radiology Tokyo Dental College Ichikawa General Hospital Chiba Japan; ^3^ Department of Internal Medicine Tokyo Dental College Ichikawa General Hospital Chiba Japan

**Keywords:** chest, CT, MRI, radiology, surfers' knots

## Abstract

A history of frequent surfing can be a key finding when a patient comes in with subcutaneous lesions on bilateral anterior lower chest. MR imaging could lead to the diagnosis with its characteristic finding for collagenous mass lesions, though most cases do not require imaging unless with atypical presentation. This enables clinicians to avoid unnecessary invasive procedures.

A 43‐year‐old male presented with painful bilateral chest lesions started a few months prior. The lesions were located anterior lower chest and painful during surfing. While his medical and family histories were unremarkable, his personal history revealed that he was a surfer with 20 years’ of experience. Neither palpation nor CT images (Figure 1), which showed soft tissue density with ill‐defined margin, reached the diagnosis. T2‐weighted and T1‐weighted MR imaging (Figure 2) showed the lesions with heterogeneous low signal intensity, with fibrous‐rich component especially with T2‐weighted imaging. These MR imaging findings along with the history of frequent surfing gave out the correct diagnosis of surfer's knots. Surfer's knots are benign acquired fibrotic connective tissue nodules that develop in response to repetitive low‐grade trauma.^1^ In our case, the patient's chest was presumably exposed to repetitive contact with a surfboard during paddling (Figure 3). They are typically seen in tibial tuberosities, dorsum of the feet, and the chest.^1^ Most cases improve by cessation of surfing. Its diagnosis does not require imaging if the presentation is typical and the clinician is aware of this condition. The radiological evaluation leads to the definitive diagnosis and enables us to avoid unnecessary invasive procedures. Sometimes the lesions remain with infection and/or pain, and may require surgical resection.^2^ If resection or detailed pretreatment evaluation is needed, CT and MR imaging are useful. Pathologically, surfer's knots are the thickening of reticular dermis as a result of increased deposition of collagen, which corresponds well with low intensity on T2‐weighted MR imaging.

**Figure 1:**
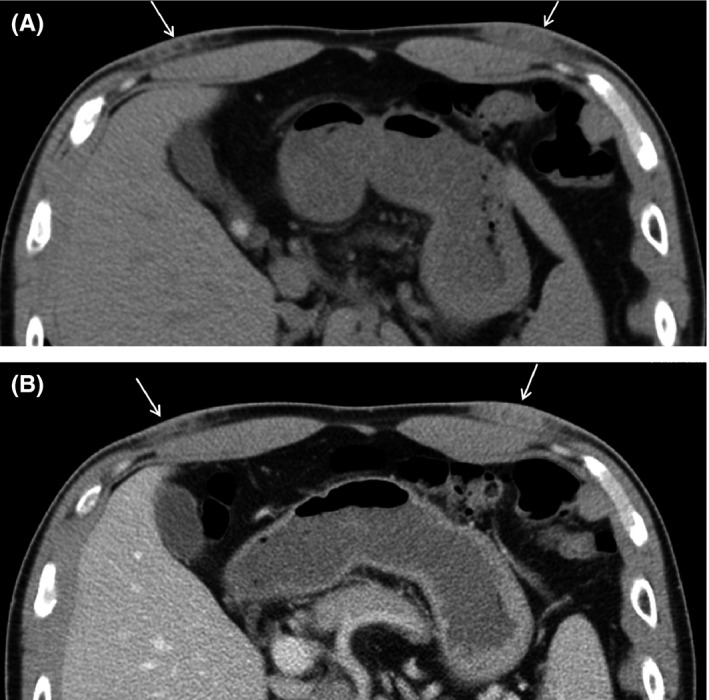
Plain CT (A) and contrast‐enhanced CT (B) revealed soft tissue density mass lesion with ill‐defined margin in subcutaneous region of bilateral anterior chest (arrows)

**Figure 2:**
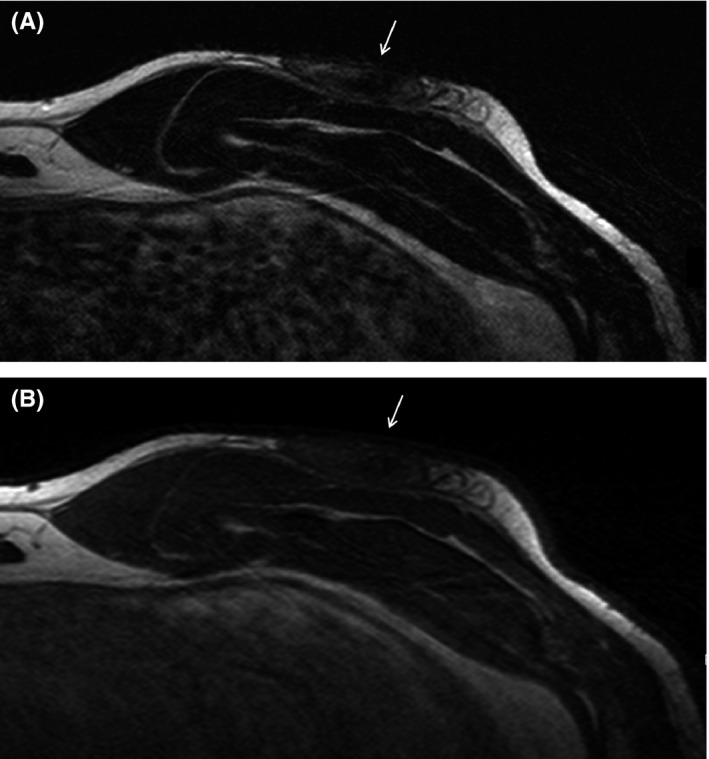
On T2‐weighted MR imaging (A) and T1‐weighted imaging (B), the lesion was identified as heterogeneous low signal intensity mass (arrows)

**Figure 3:**
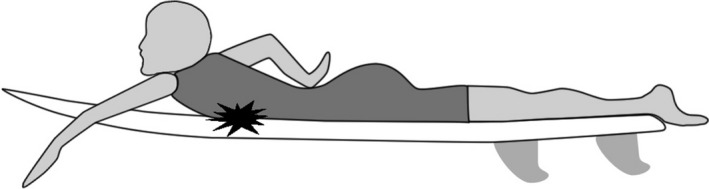
Schematic drawing of the contact points of a surfer's chest to a surfboard when paddling

## CONFLICT OF INTEREST

None declared.

## AUTHOR CONTRIBUTIONS

AB: drafted the article. All authors participated in critical review and the revision of the articles. All authors gave the final approval of the article. All authors have accountability for all aspects of the work.
